# Infection and nuclear interaction in mammalian cells by ‘*Candidatus* Berkiella cookevillensis’, a novel bacterium isolated from amoebae

**DOI:** 10.1186/s12866-019-1457-z

**Published:** 2019-05-09

**Authors:** Nicholas B. Chamberlain, Yohannes T. Mehari, B. Jason Hayes, Colleen M. Roden, Destaalem T. Kidane, Andrew J. Swehla, Mario A. Lorenzana-DeWitt, Anthony L. Farone, John H. Gunderson, Sharon G. Berk, Mary B. Farone

**Affiliations:** 1 Molecular Biosciences Program, Middle Tennessee State University, 1301 E. Main St, Murfreesboro, TN 37130 USA; 2 Department of Biology, Middle Tennessee State University, 1301 E. Main St, Murfreesboro, TN 37130 USA; 3 Department of Biology, Tennessee Technological University, 1 William L Jones Dr, Cookeville, TN 38505 USA

**Keywords:** Bacteria, Nucleus, *Coxiella*, *Legionella*, Human, Symbiosis, Endosymbiont

## Abstract

**Background:**

‘*Candidatus* Berkiella cookevillensis’ and ‘*Ca.* Berkiella aquae’ have previously been described as intranuclear bacteria of amoebae. Both bacteria were isolated from amoebae and were described as appearing within the nuclei of *Acanthamoeba polyphaga* and ultimately lysing their host cells within 4 days. Both bacteria are Gammaproteobacteria in the order *Legionellales* with the greatest similarity to *Coxiella burnetii.* Neither bacterium grows axenically in artificial culture media. In this study, we further characterized ‘*Ca.* B. cookevillensis’ by demonstrating association with nuclei of human phagocytic and nonphagocytic cell lines.

**Results:**

Transmission electron microscopy (TEM) and confocal microscopy were used to confirm nuclear co-localization of ‘*Ca.* B. cookevillensis’ in the amoeba host *A. polyphaga* with 100% of cells having bacteria co-localized with host nuclei by 48 h. TEM and confocal microscopy demonstrated that the bacterium was also observed to be closely associated with nuclei of human U937 and THP-1 differentiated macrophage cell lines and nonphagocytic HeLa human epithelial-like cells. Immunofluorescent staining revealed that the bacteria-containing vacuole invaginates the nuclear membranes and appears to cross from the cytoplasm into the nucleus as an intact vacuole.

**Conclusion:**

Results of this study indicate that a novel coccoid bacterium isolated from amoebae can infect human cell lines by associating with the host cell nuclei, either by crossing the nuclear membranes or by deeply invaginating the nuclear membranes. When associated with the nuclei, the bacteria appear to be bound within a vacuole and replicate to high numbers by 48 h. We believe this is the first report of such a process involving bacteria and human cell lines.

**Electronic supplementary material:**

The online version of this article (10.1186/s12866-019-1457-z) contains supplementary material, which is available to authorized users.

## Background

‘*Candidatus* Berkiella cookevillensis’ has recently been described as a novel bacterium isolated from an amoeba in a biofilm sample from a cooling tower [1, 2]. This bacterium and the closely related bacterium, ‘*Ca.* Berkiella aquae’, have been assigned to the order *Legionellales* within the Gammaproteobacteria. They most closely resemble members of the genera *Coxiella* and *Legionella* [1]. Both of these novel bacterial strains appear to invade the nuclei of their amoebal hosts. Bacterial invasion of the nucleus has been described for multiple protozoan species, most notably in the ciliate genus *Paramecium*, but also for flagellates and free-living amoebae [3]. Such intranuclear invasion by bacterial symbionts can result in a range of host responses from a mutualistic or commensal association, as has been described for ‘*Ca.* Nucleicultrix amoebiphila’ and its *Hartmanella* spp. host, to a parasitic interaction as for *Holospora* spp. that develops a specialized infectious form ultimately lysing the *Paramecium* host [4–6].

While invasion of the protozoan nucleus has been reported for several groups of bacteria, including Verrucomicrobia, Chlamydia, Alphaproteobacteria, and Gammaproteobacteria, invasion of mammalian cell nuclei has been limited to members of the Alphaproteobacteria, *Mycoplasma genitalium*, and more recently the gram-negative Betaproteobacterium, *Burkholderia pseudomallei* [3, 7–10]. Generally low frequencies of infection have been observed in cultured mammalian cells, although replication within nuclei has been reported [11–13]. For the Alphaproteobacteria and potentially *B. pseudomallei*, nuclear invasion may result from a propulsion into the nucleus via polymerized actin tails following the escape of bacteria from intracellular membrane compartments [7, 10, 11]. Protozoan intranuclear symbionts may also rely on actin for nuclear invasion. Like *Rickettsia* spp. and *B. pseudomallei*, *Holospora* spp. also exit their *Paramecium* host phagosome and may utilize an 89 kD protein located at the tip of a large periplasmic space referred to as the “invasion tip.” This protein contains actin-binding motifs that influence actin-based motility of the bacterium, and actin tails have been observed to be associated with *Holospora obtusa* entering the host macronucleus [3, 4, 14, 15]. Bacteria that have escaped the phagosome might also move to the nucleus by associating with chromatin after breakdown of the nuclear membranes during mitosis and remain with it while the nuclear membranes are reformed following mitosis. This strategy has been described for *Nucleicultrix amoebiphila* in its amoebal hosts [3]. Less information on nuclear entry is available for bacteria that do not first escape their cytoplasmic compartment. However, in *Euglena hemichromata,* it appears that bacteria-containing cytoplasmic vacuoles fuse with the outer nuclear membrane, releasing bacteria into the nuclear periplasmic space, which then subsequently invaginate the inner nuclear membrane for entry into the nucleus [16].

Despite the descriptions of intranuclear bacteria both in protozoan and mammalian cells, there have been few descriptions of bacteria that infect and replicate within the nuclei of multiple eukaryotic phyla. *Rickettsia bellii*, originally isolated from *Dermacentor variabilis* ticks, infects the nuclei of mammalian Vero and amphibian XTC-2 cells. *R. bellii* also survives within *Acanthamoeba polyphaga*, although nuclear invasion in the amoebae was not described [11, 17]. The present study is an examination of the recently described intracellular amoebal pathogen, ‘*Ca.* B. cookevillensis,’ with the nuclei of human cell lines and the entry of vacuoles containing this bacterium into the nuclei of host cells.

## Results

### ‘*Ca.* Berkiella cookevillensis’ invades the nucleus of *Acanthamoeba polyphaga*

**Fig. 1:**
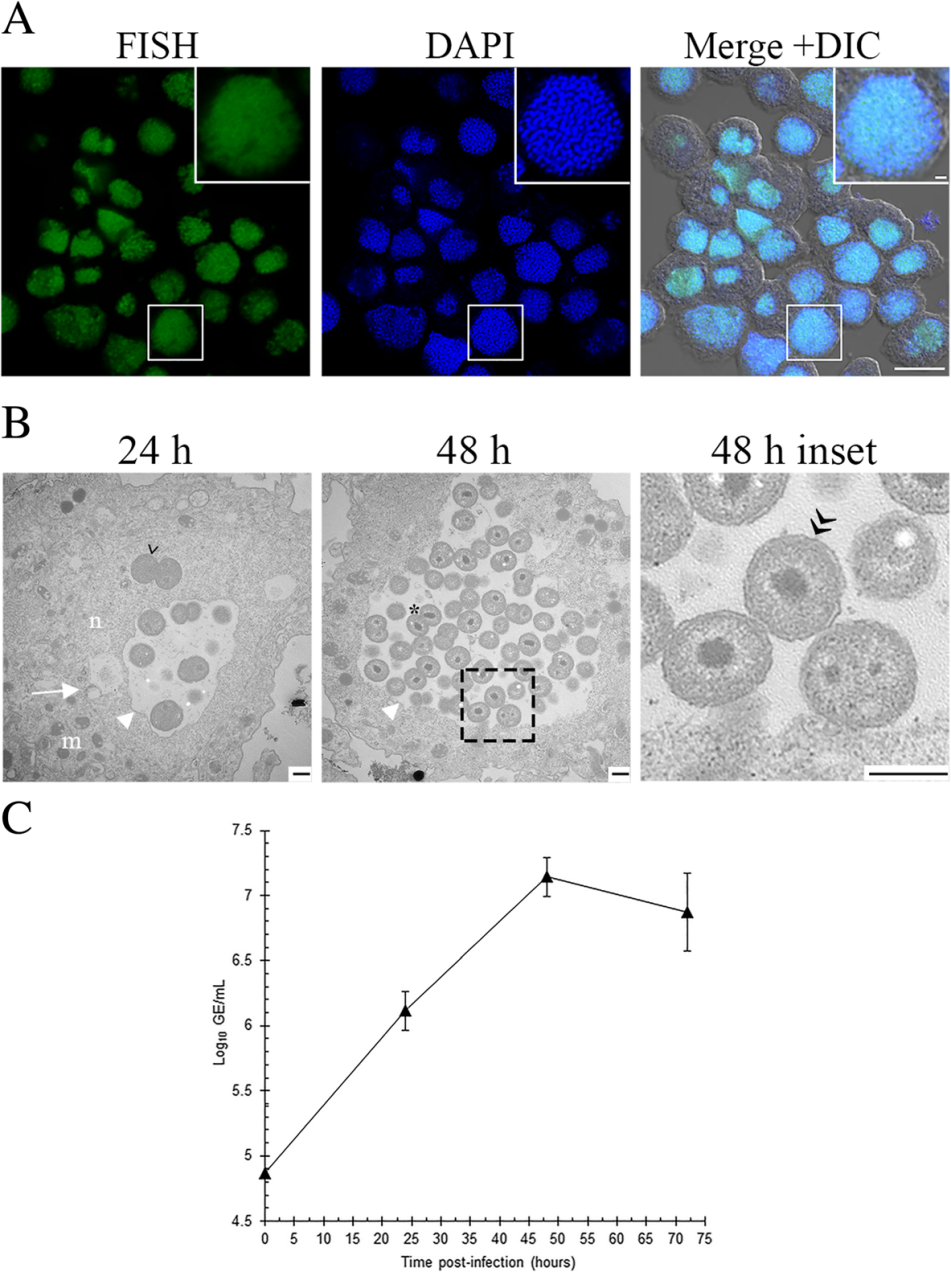
Growth of ‘*Ca.* Berkiella cookevillensis’ in *Acanthamoeba polyphaga. A. polyphaga* was infected for 48 h with ‘*Ca.* B. cookevillensis’ at an MOI of 1. **a** Intracellular bacteria were detected using confocal microscopy with helper and 6-FAM-labeled FISH probes specific for the 16S rRNA of the bacterium (green). DAPI staining was used to visualize the amoebal nucleus but also stains the bacteria (blue). Large numbers of bacteria are co-localized with nuclei of amoebae by 48 h p.i. Bars, 10 μm. **b** TEMs showing the growth of ‘Ca. B. cookevillensis’ following infection for 24 and 48 h in *A. polyphaga* at an MOI of 1. At 24 h, small numbers of bacteria are visible within the double-membraned (white arrow) nucleus (n). The euchromatin of the nucleoplasm appears more electron-lucent than the cytoplasm. Mitochondria (m) are visible in the cytoplasm. The bacteria are most often seen within an electron-translucent space surrounded by a darkened, single membrane (white triangle). More than one bacteria-containing vacuole often appears within a nucleus. On occasion, no electron-translucent space or membrane is visualized around the bacteria, as for the bacterium at 24 h (indicated by >). By 48 h, increased numbers of bacteria are within bacteria-containing vacuoles and dividing bacteria are evident (*). The gram-negative outer membrane can also be distinguished in TEMs as is indicated in the magnified insets at 48 h (>>). Bars, 500 nm. **c** Bacterial growth of ‘Ca. B. cookevillensis’ in *A. polyphaga* as confirmed by qPCR. ‘Ca. B. cookevillensis’ increased by 1.5 log_10_ when infected at an MOI of 1. Data represent means of two independent experiments performed in triplicate


We have recently described an intracellular endosymbiont isolated from an amoeba, ‘*Ca.* B. cookevillensis’ [1, 2]. The bacterium was isolated from an amoeba found in a cooling tower, and is a coccoid bacterium of 1–3 μm diameter [1]. FISH was used to confirm the identity of the intracellular bacterium in *A. polyphaga* (Fig. 1a). Because the unique sequences for ‘*Ca*. Berkiella cookevillensis’ were located in the class V and VI regions of the rRNA, which have low accessibility to in situ probes, helper probes were used to increase the signal [18]. TEM shows that the endosymbiont is enclosed in a bacteria-containing vacuole (BCV) and appears to be within the euchromatin network of the nucleus (Fig. 1b). The bacteria are surrounded in the vacuole by electron-translucent space, which may indicate production of extracellular polysaccharide. Occasionally, bacteria were observed to be without an obvious surrounding vacuole or extracellular matrix (Fig. 1b 24 h) indicating the potential for more than one mode of infection. TEM also confirms the gram-negative cell wall structure (Fig. 1b 48 h inset). Evidence of dividing cells was observed for these intracellular bacteria, and increases in numbers of bacteria were also evident over a 48 h time period (Fig. 1b, Additional file 1: Figure S1A). Bacterial-specific qPCR showed that bacteria increased by 1.5 log_10_ over a 72 h period (Fig. 1c). By 48 and 72 h p.i., greater than 90% of the amoebal cells had BCVs co-localized with amoeba nuclei (Additional file 1: Figure S1A). After 72 h, most host cells had lysed or were too fragile to stain properly. Observations of the bacteria, reveal that before 48 h, bacteria appear nonmotile (Additional file 2, part A), but by 48 h, a “jiggling” movement of bacteria within BCVs can be detected (Additional file 2, part B), which progresses to rapid, circular movement by 72 h (Additional file 2, part C). The bacteria remained motile in the infection buffer for several hours following host cell lysis, after which the bacterial cells became nonmotile and adhered to the bottom of culture flasks or plates, presumably facilitating their engulfment by grazing amoebae. The vacuolar electron-translucent space visible in TEMs suggests the presence of extracellular matrix material which might contribute to the biofilm-like arrangement of the bacteria adhering to the bottom of culture flasks. In all TEM sections of ‘*Ca.* Berkiella cookevillensis’-infected *A. polyphaga* we observed the intranuclear appearance of a BCV; however, given the two-dimensional nature of TEM sections, the possibility that these images depict the deep invagination of a BCV into the nuclear regions, as with a fist depressed into a slightly underinflated balloon, cannot be eliminated.

**Additional file 2:** Video (mp4 file) of bacterial movement within *A. polyphaga* at 1000X magnification using an Olympus BX60 microscope with DP72 camera and cellSens imaging software. (MP4 9634 kb)


### ‘*Ca.* Berkiella cookevillensis’ invades nuclei of phagocytic human cell lines

**Fig. 2:**
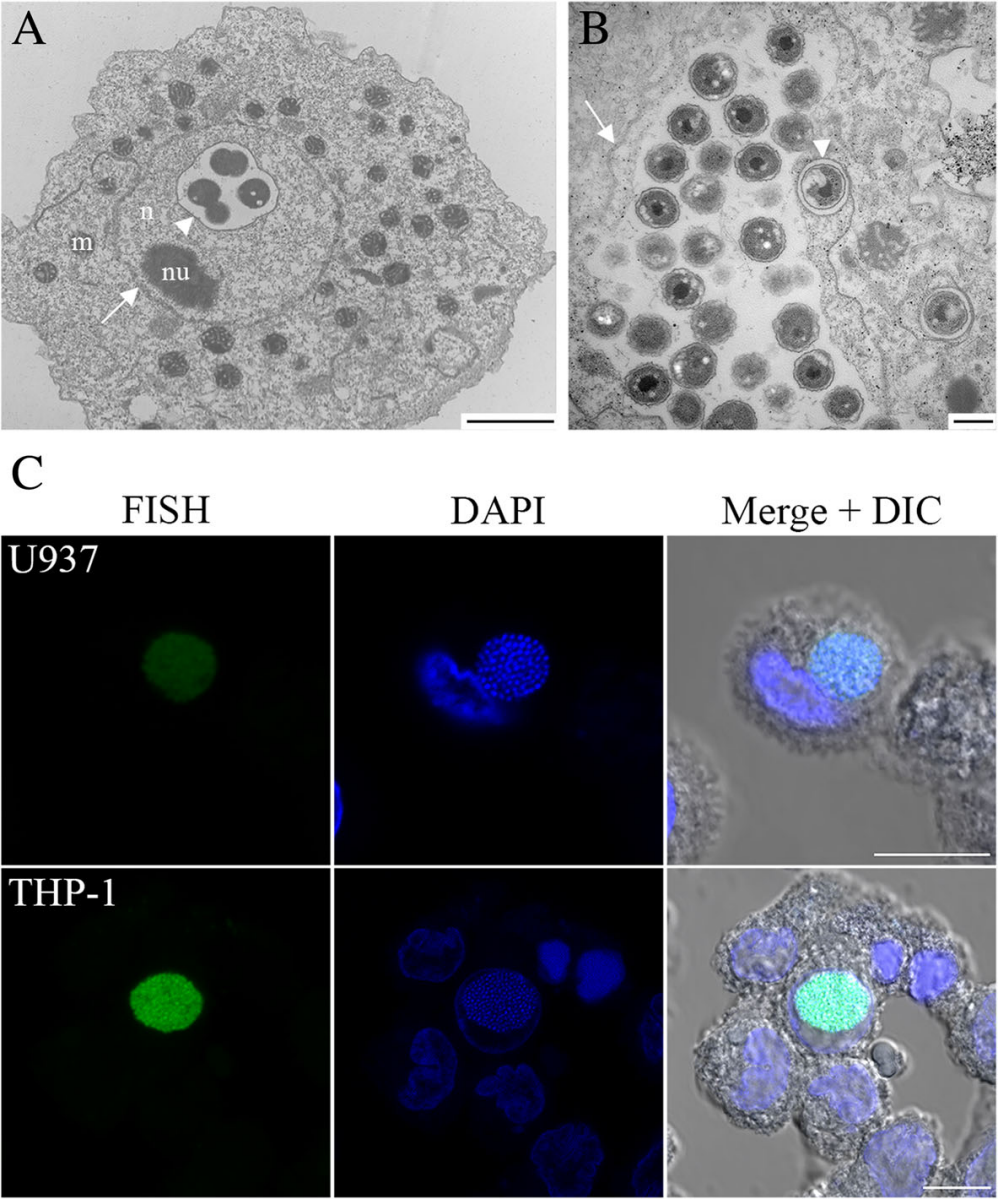
Growth of ‘*Ca.* B. cookevillensis’ in human macrophage-like cells. TEMs of ‘Ca. B. cookevillensis’ appearing to be within the nucleus (n) of human U937 macrophages at 24 h (**a**) and 48 h (**b**) following infection with an MOI of 10. At 24 h, bacteria are contained within the nucleoplasm surrounded by electron-translucent space within a vacuole (white triangle). The bilayer of the nuclear envelope is visible (white arrow). The nucleolus (nu) is also visible. By 48 h, larger numbers of bacteria are visible in the nuclear vacuoles and the vacuole fills the nucleus. A cytoplasmic vacuole with a single bacterium is also visible. **c** Confocal micrographs of FISH stains using probes specific for ‘Ca. B. cookevillensis’ in U937 cells (top) and an additional human macrophage cell line, THP-1 (bottom) at 48 h p.i. Nuclei and bacteria are also stained with DAPI. Inclusions filled with bacteria are visible for both cell lines. Bars, 10 μm

**Fig. 3:**
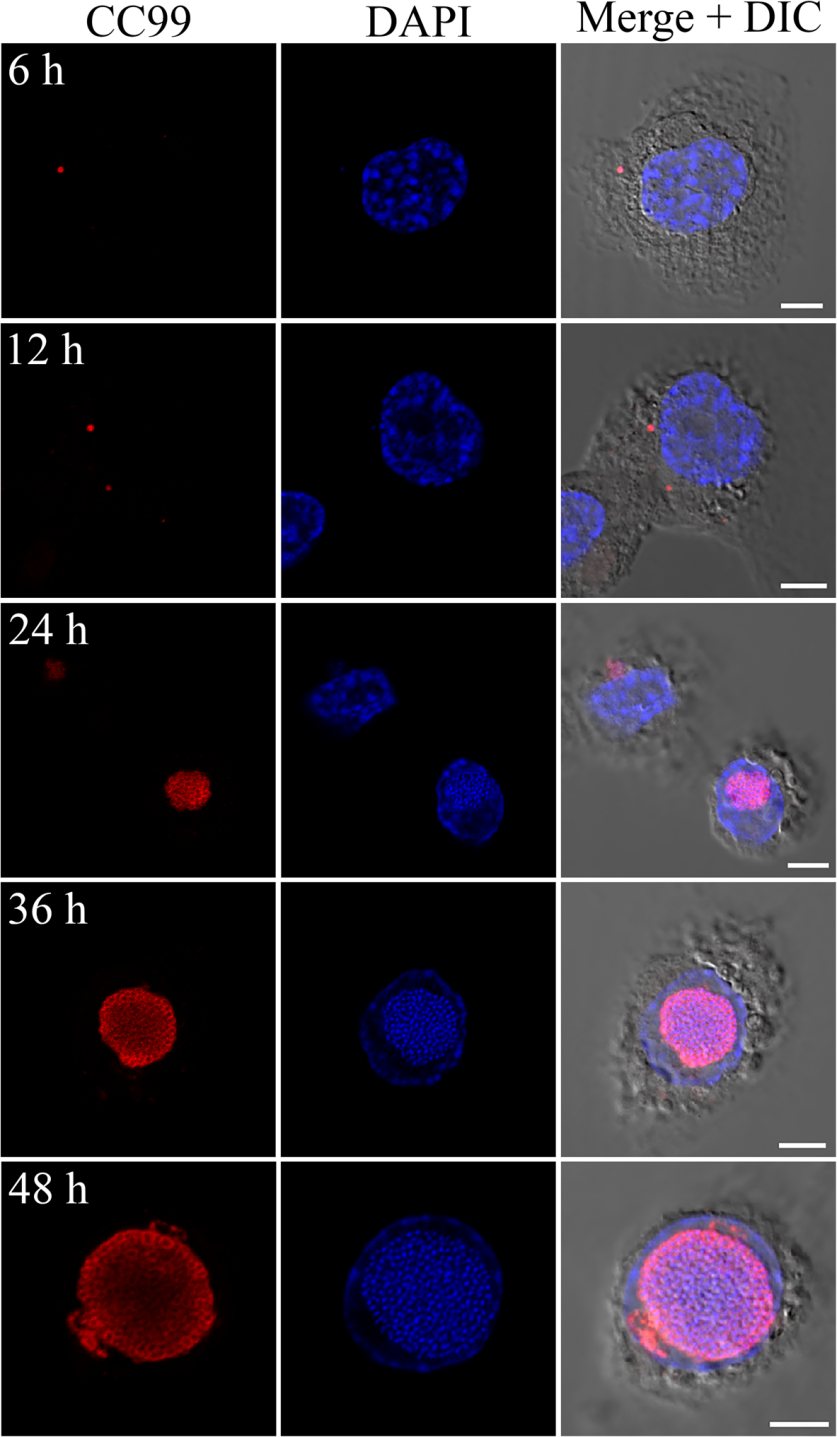
Time course of ‘*Ca.* B. cookevillensis’ infection in PMA-differentiated THP-1 macrophage-like cells. THP-1 cells were infected with ‘Ca. B. cookevillensis’ (CC99) for 1 h at an MOI of 10 and incubated for the designated amount of time. Following incubation, immunofluorescent staining of bacteria was conducted with rabbit anti-serum to ‘Ca. B. cookevillensis’ and a TRITC-conjugated secondary antibody prior to DAPI staining. At 6 h p.i., puncta of individual or small clusters of bacteria are visible in the cytoplasm. By 12–24 h, the bacterial inclusions appear perinuclear or co-localized with the nucleus. At 36–48 h, the bacteria-containing vacuoles are filled with bacteria, and appear to be enclosed within the nuclear envelope. Imaging was performed with laser scanning confocal microscopy. Bars, 5 μm


When PMA-differentiated U937 human macrophage-like cells were treated with ‘*Ca.* B. cookevillensis’ at an MOI of 10, bacteria co-localizing with host nuclei were visible in TEM images by 24 h p.i. and were enclosed in a vacuole within the euchromatin. As with the amoebae, an electron-translucent space surrounds the individual cells with evidence of dividing cells and an increased number of bacteria by 48 h (Fig. 2a-b). FISH confirmed the bacteria as ‘Ca. B. cookevillensis’ in both U937 and THP-1 human macrophage-like cell lines (Fig. 2c). A time course of infection in THP-1 cells using indirect immunofluorescent staining to detect ‘Ca. B. cookevillensis’ showed that by 6 h p.i., discrete puncta of bacteria could be detected in the cytoplasm, which by 12 h were perinuclear (Fig. 3). By 24 h, larger inclusions of bacteria, indicative of replication, were perinuclear or co-localized with the nucleus (Fig. 3). At 36–48 h p.i., large vacuoles of bacteria were either co-localized with nuclei or appeared to be within nuclei. The progressive increase in BCV size is indicative of bacterial replication within the vacuoles (Fig. 3, 36–48 h). A confocal Z-stack of BacLight-stained bacteria in U937 cells also shows their location in the stained nucleus at 48 h p.i. (Additional file 3). Enumeration of U937 cells treated with ‘Ca. B. cookevillensis’ showed patterns of infection similar to those detected in THP-1 cells (Fig. 3) with 32% of cells infected and nearly 20% of cells having BCVs co-localized with nuclei at 24 h (Additional file 1: Figure S1B). Between 48 and 72 h p.i., infection levels in U937 cells increased from 39 to 55% with up to 42% of cells having BCVs co-localized with nuclei; however, there were no significant differences between cells with intracellular bacteria and cells with nuclear-associated vacuoles, indicating that by this time, almost all BCVs were nuclear localized (Additional file 1: Figure S1B). However, we also observed large cytoplasmic BCVs not associated with nucleus, suggesting that the nucleus is not a requisite for growth. After 72 h, significant cell lysis occurs so that very few intact host cells were detectable by 96 h p.i. Growth of ‘Ca. B. cookevillensis’ in macrophage-like cells resulted in a 1.25 log_10_ increase over a 96-h period with the greatest increase occurring from 0 to 48 h p.i. (1.15 log_10_) (Additional file 1: Figure S1C). ‘Ca. B. cookevillensis’ also infected and appeared to be intranuclear in murine macrophage-like RAW 264.7 cells, showing its broader host diversity (Additional file 1: Figure S2).

**Additional file 3:** Confocal Z-stack video (mp4 file) of bacteria stained with BacLight in a U937 cell nucleus. (MP4 393 kb)


### ‘*Ca.* Berkiella cookevillensis’ invades nuclei of nonphagocytic cells

**Fig. 4:**
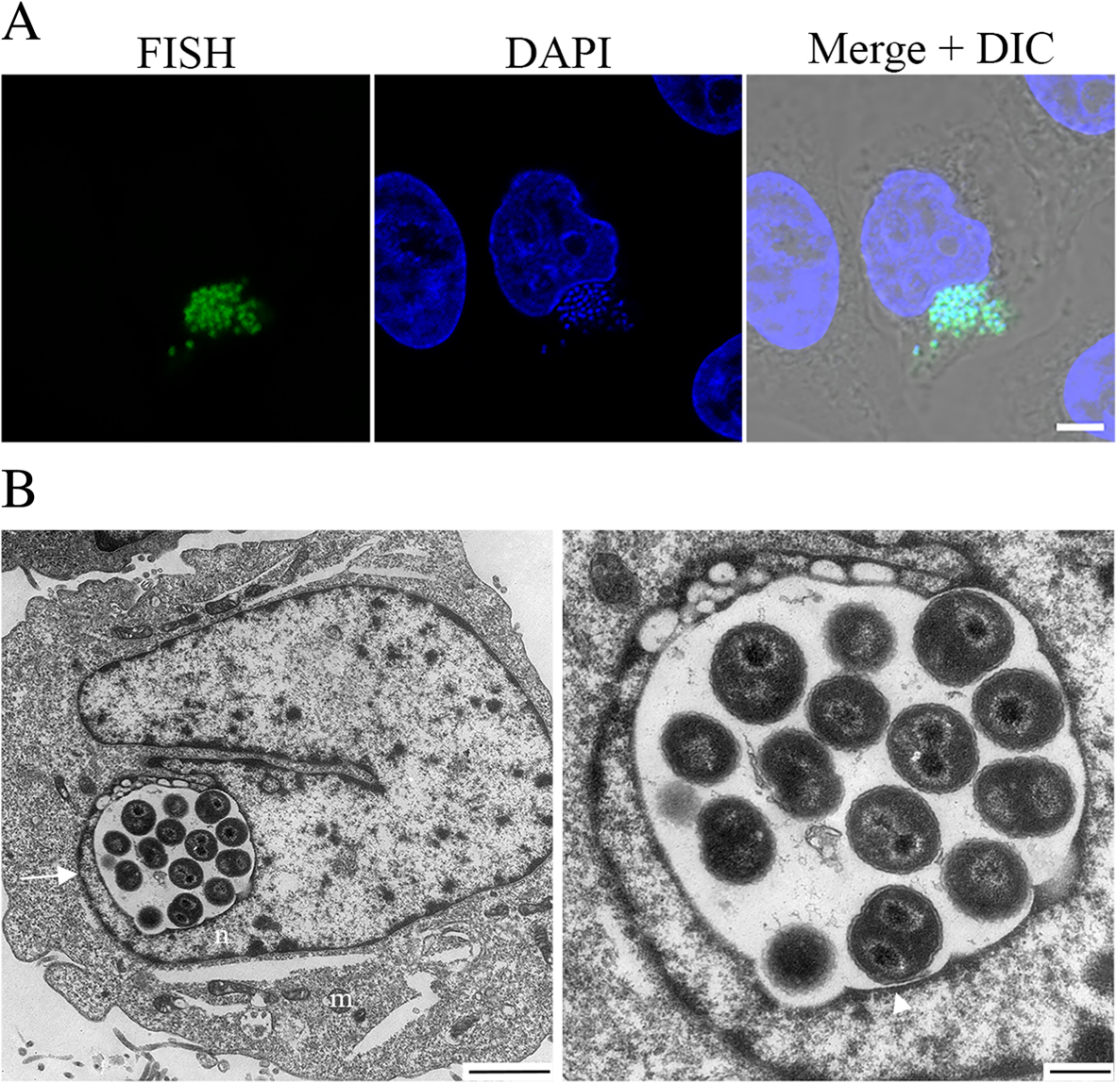
Growth of ‘*Ca.* Berkiella cookevillensis’ in non-phagocytic HeLa cells. **a** Confocal micrographs of FISH staining with 6-FAM-conjugated probe and helper probes targeting the 16S rRNA of ‘*Ca.* B. cookevillensis’ confirms the infectivity of ‘Ca. B. cookevillensis’ for HeLa human epithelial-like cells. Bar, 5 μm. **b** TEM images of bacteria appearing to be within the nucleus (n) of a HeLa cell. The left image (bar, 2 μm) shows the location of the nuclear envelope (white arrow). Mitochondria (m) are visible in the cytoplasm. The bacteria appear surrounded by electron-translucent space within a vacuole. The vacuolar membrane (white triangle) is visible in the enlarged image (right) with the vacuole surrounded by loose euchromatin. Dividing cells can also be seen in the image (bar, 500 nm)

**Fig. 5:**
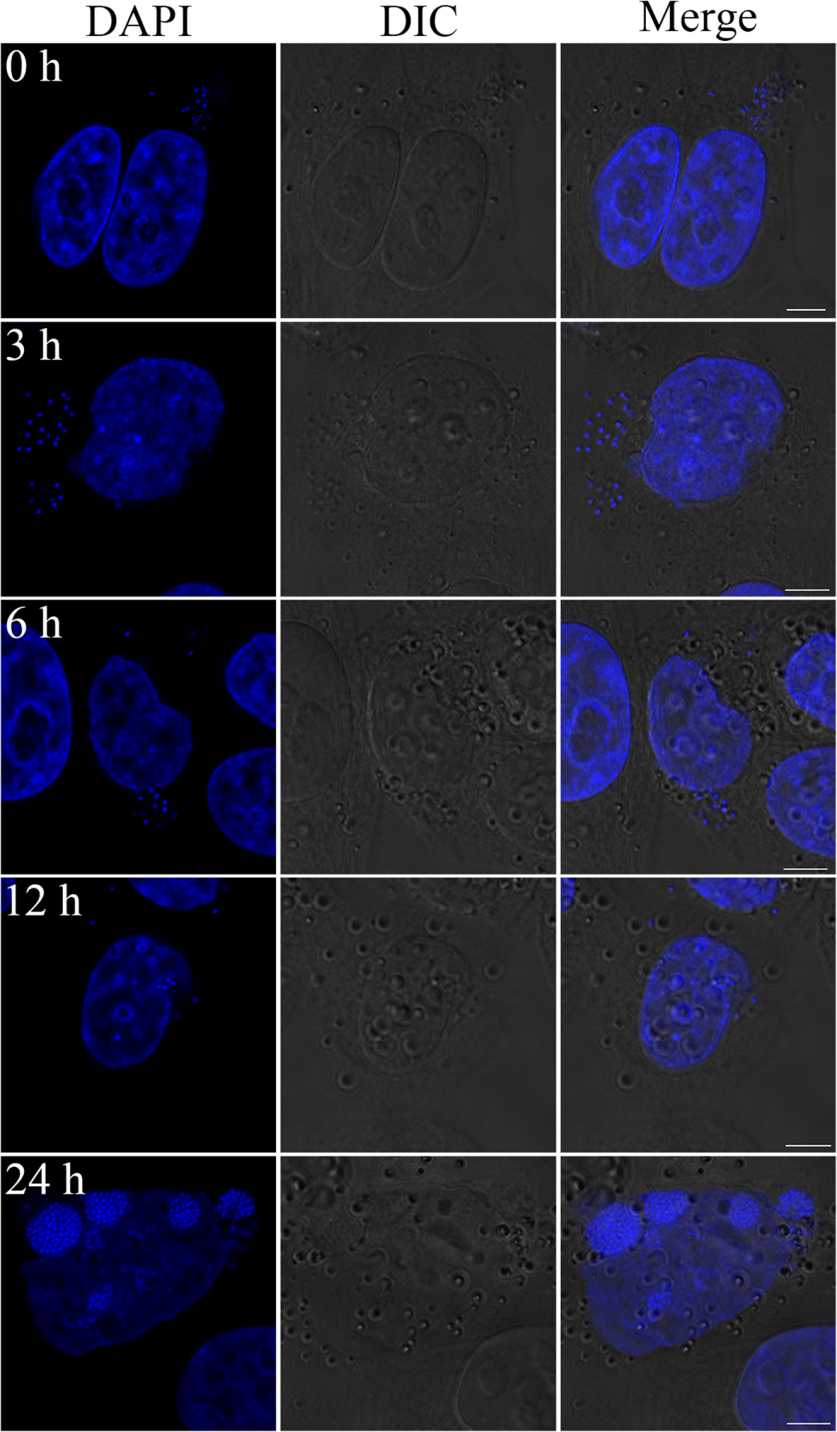
Time course of ‘*Ca.* Berkiella cookevillensis’ infection in HeLa cells. HeLa cells were infected with ‘*Ca.* B. cookevillensis’ for 1 h at an MOI of 200, and then incubated for the designated amount of time. Following the 1 h incubation (time 0), bacterial and mammalian DNA was stained with DAPI. Imaging was performed with laser scanning confocal microscopy. After only 1 h of incubation, distinct bacteria can be found in the cytoplasm. By 3 h p.i., bacteria are still within the cytoplasm, but by 6–12 h, bacteria are more perinuclear. At 24 h, larger numbers of bacteria are present within cytoplasmic vacuoles and nuclear-associated vacuoles. Bars, 5 μm

‘*Ca.* B. cookevillensis’ was also intracellular for human epithelial-like HeLa cells as confirmed by FISH (Fig. 4a), and as described above for both amoebae and macrophages, bacteria in the nucleus are surrounded by translucent space in a BCV within the euchromatin (Fig. 4b). The inset for Fig. 4b highlights the single membrane surrounding the bacteria; however, the upper right quadrant of the image depicts a very close association between the nuclear and BCV membranes. Because the TEM image is two dimensional, it is difficult to assess whether this represents a bacterial vacuole invaginated into the nucleus or one which is truly inside the nucleus. Bacteria-infected HeLa cells stained with DAPI in a time course study showed low numbers of bacteria in the cytoplasm from 1 h (time 0) until 12 h p.i (Fig. 5). By 24 h, increased numbers of bacteria were in the cytoplasm clustered in discrete packets, indicative of replicating cells (Fig. 5). By this time point, indentations of the nuclear membranes by perinuclear inclusions were again evident, and multiple BCVs appear to cover the nuclear membranes, although 5.6% of total cells have intracellular bacteria with only 3.7% of nuclei appearing to have intranuclear inclusions, which was significantly lower than for U937 cell infections (Additional file 1: Figure S1B).

From 48 to 72 h p.i., infection rates increased with 46% of total cells infected and 38% of cells with nuclear co-localization of BCVs. The percentages of infected nuclei for HeLa cells were not significantly different from U937 cells at 48 and 72 h p.i., although both U937 and HeLa cells exhibited lower levels of infection than did *A. polyphaga* (Additional file 1: Figure S1B). Similar to the macrophage-like cells, large cytoplasmic BCVs were also observed. By 96 h of infection, lysis of many of the HeLa cells had occurred. However, we did observe that the infected HeLa cells tended to persist longer in culture. This is likely due to the continued proliferation of HeLa cells, whereas differentiated THP-1 or U937 cells no longer proliferate. As a result, a more persistent infection occurred in the HeLa cells as new host cells were produced for the bacteria. Real-time qPCR showed that ‘*Ca.* B. cookevillensis’ exhibited a 1.35 log_10_ increase by 96 h p.i. (Additional file 1: Figure S1C). Bacteria from HeLa or macrophage-like cells were infectious for the reciprocal cell types and *A. polyphaga.*

### Membranes of intranuclear ‘*Ca.* Berkiella cookevillensis’ bacteria-containing vacuoles do not contain nuclear lamin or nuclear pore protein

**Fig. 6:**
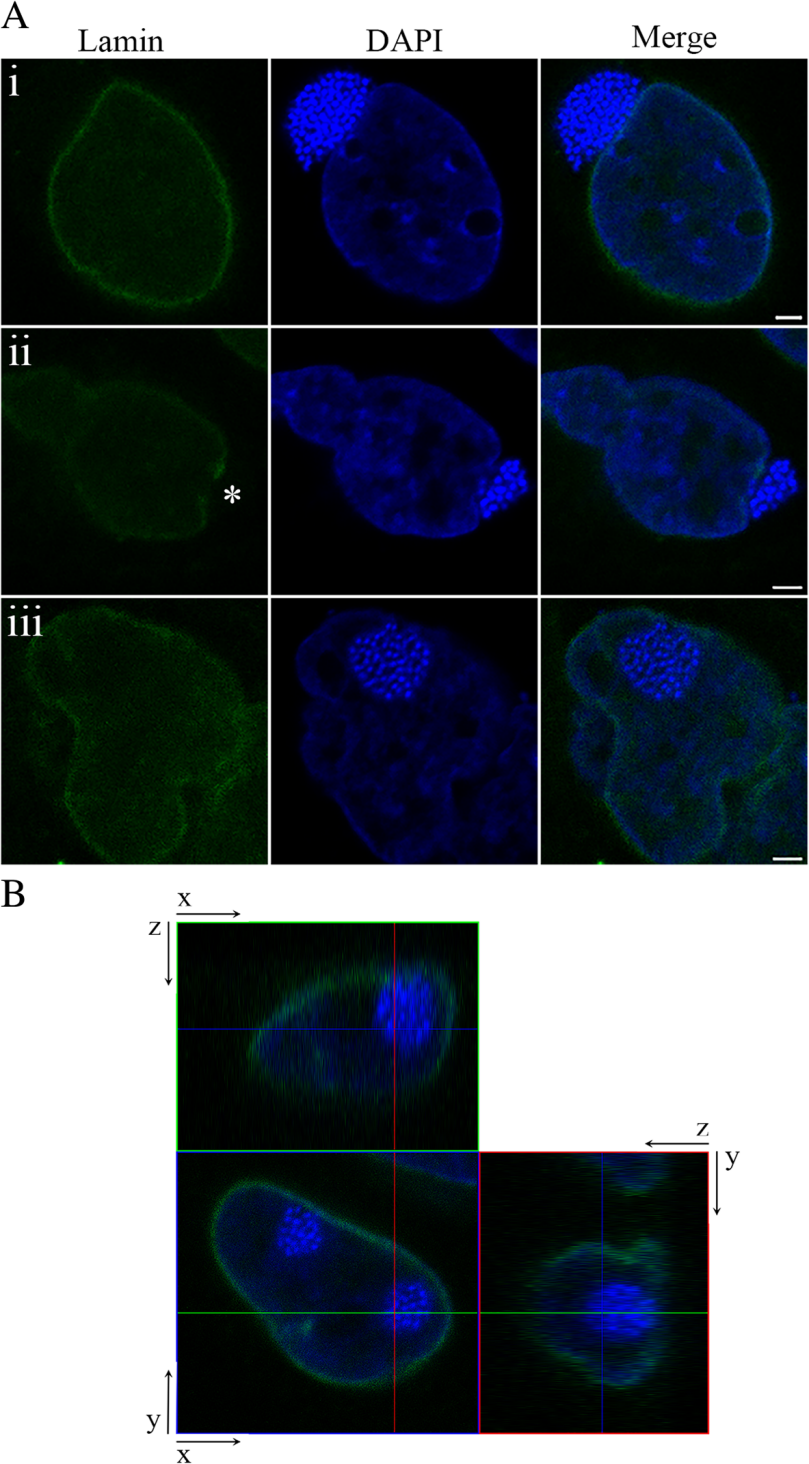
Lamin staining of ‘*Ca.* Berkiella cookevillensis’-treated HeLa cells. Indirect immunofluorescent staining of lamin A/C (green) performed in conjunction with a DAPI stain (blue) for HeLa cell and bacterial DNA. **a** Representative confocal micrographs of HeLa cells infected with ‘*Ca.* B. cookevillensis’ at an MOI of 200 for 24 h demonstrate the invasion process of bacteria-containing vacuoles into the host nucleus (i-iii). (i) Image depicts the bacteria-containing vacuole localized to the juxtanuclear region of the HeLa cell with lamin staining indicating an intact nuclear lamina. (ii) Entry of the ‘Ca. B. cookevillensis’ vacuole into the nucleus. Asterisk (*) denotes site of nucleus invasion with a disruption of lamin staining. (iii) Intact bacteria-containing vacuole within the nucleus of the host cell with no indication of lamin staining of the ‘Ca. B. cookevillensis’ vacuole. Bars, 2 μm. **b** z-y and z-x projections demonstrate the bacteria-containing vacuole within the nucleus


By 12–24 h p.i., ‘*Ca.* B. cookevillensis’ is contained within BCVs both in the cytoplasm and either co-localized or within the nucleus of its host cells. From observations of immunofluorescent stains and TEMs, we noticed repeatedly that the juxtanuclear inclusions of bacteria induce an indentation of the nuclear membranes (Fig. 3, 48 h and Fig. 5, 24 h). To further investigate nuclear invasion by ‘*Ca.* B. cookevillensis’, we performed indirect immunofluorescent staining with antibodies for nuclear lamin A/C. Fig. 6 depicts HeLa cells with both perinuclear and intranuclear inclusions of ‘Ca. B. cookevillensis’ at 24 h p.i. The juxtanuclear BCV and the indentation of the nuclear envelope (Fig. 6ai) suggest that the bacteria-rich vacuole applies a mechanical force or induces ruffling of the nuclear envelope. The HeLa cell in the 24 h view of Fig. 5 has lateral inclusions of bacteria as well as BCVs visible in the top view of the cell. Confocal imaging with lamin A/C staining (Additional file 4) shows that the BCV on the left side of the cell is invaginated into the nuclear area, as indicated by the ring of stained lamin that surrounds the bacteria, as would occur with the “fist in a balloon” scenario mentioned above. However, the BCV near the top right of the cell in Fig. 5 (24 h; Additional file 4) is not encircled by nuclear lamina indicating its location within the nucleoplasm. Furthermore, it appears that the entire BCV is capable of entering the host nucleus through a break in the nuclear lamina (Fig. 6aii, Additional file 4). Breaks in the lamina can be detected only in areas where there is a juxtanuclear BCV. During and following entry of the vacuole into the nucleus, the nuclear lamina does not encapsulate the BCV nor does the vacuole acquire significant amount of the lamina as it does not stain positively for lamin (Fig. 6aii and aiii). Since the nuclear lamina is tethered to the inner nuclear membrane, this suggests that the inner nuclear membrane does not associate with the BCV. Moreover, the bacteria are maintained within a vacuole through the entirety of the nuclear invasion process (Fig. 6aii and aiii). Additionally, TEMs do not indicate more than a single membrane surrounding intranuclear BCVs (Figs. 1b, 3a, 4b). Upon the successful nuclear infiltration of the BCV, the invasion-induced nuclear lamina lesion appears to be ameliorated and no protrusion or release of chromatin was detected at the site of nuclear entry (Fig. 6aiii). These observations indicate that the ‘*Ca.* B. cookevillensis’-rich vacuole does not invade the nucleus via a method that causes general degradation of the nuclear envelope, but instead implies a regulated entry into the nucleus.

**Additional file 4:** Confocal Z-stack video (mp4 file) of bacteria in HeLa cells stained with DAPI and anti-lamin A/C antibody, followed by the Z-stack with lamin immunostaining only. (MP4 16557 kb)


To confirm that the BCV was within the nucleus, we created a Z-stack of ‘*Ca.* B. cookevillensis’-infected HeLa cells that were co-stained for lamin immunofluorescence and DAPI. The orthogonal view of the Z-stack projection shows the BCV enclosed by the nuclear lamina of the host cell (Fig. 6b), thus confirming that the vacuole is inside the host cell nucleus. The entry of ‘*Ca.* B. cookevillensis’ BCVs into the nucleus is not an individual event, as multiple inclusions within a single nucleus have been observed, yet the nuclear envelope remains intact (Fig. 6b).

**Fig. 7:**
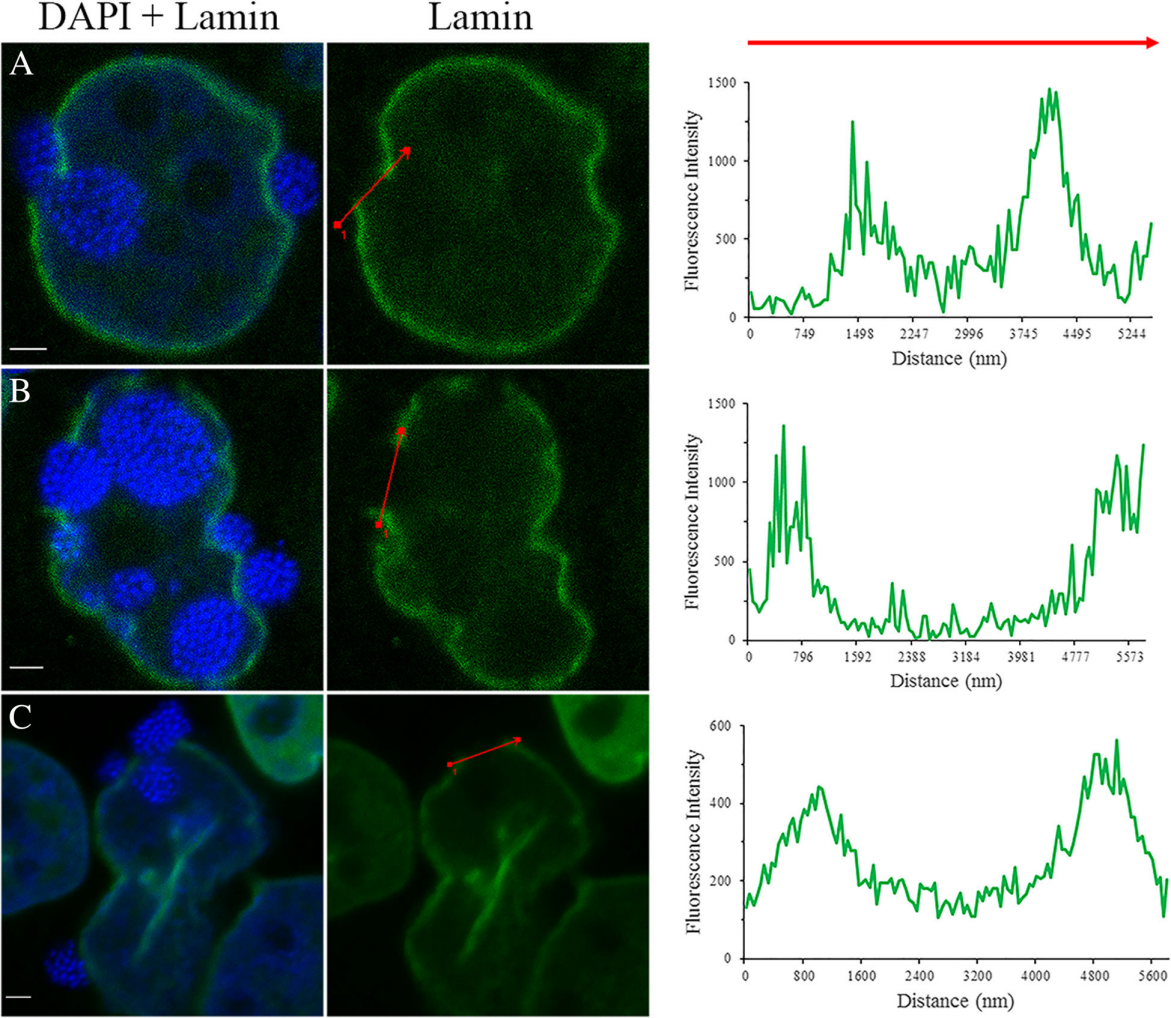
Detection of lamina gaps in nuclei of ‘*Ca.* Berkiella cookevillensis’-treated HeLa cells. Indirect immunofluorescence staining of HeLa cells infected with ‘*Ca*. B. cookevillensis (CC99) at an MOI of 200 for 24 h. HeLa cells were labeled using an anti-lamin A/C antibody and co-stained with DAPI. **a-c** Three examples of CC99-rich vacuoles invading the host cell nucleus. The DAPI + lamin merged micrograph demonstrates the position of bacterial DNA to the nuclear lamina. The fluorescence intensity profile for the lamin stain was constructed to quantify the size of the nuclear lamina gap (red arrow). Bars, 2 μm

In confocal images, the appearance of an intact BCV squeezing through the nuclear lamina can be observed (Fig. 7a, Additional file 5). To further analyze the nuclear lamina gap distance formed during the vacuole invasion, ZEN 2012 software was used to obtain a fluorescence profile across the lamina gap. The fluorescence profiles obtained for three examples demonstrate lamina gap distances of approximately 2000 nm (Fig. 7a), 2750 nm (Fig. 7b), and 4250 nm (Fig. 7c). These nuclear lamina gap distances formed during ‘*Ca.* B. cookevillensis’-vacuole invasion of the nucleus are well beyond the maximum 39 nm calculated diameter of the nuclear pore complex [19]. These results suggest that this bacterium utilizes a method other than passing through the nuclear pore complex for entry of the BCV into the host cell nucleus. The gap distances are also greater than the estimated size of 1-3 μm for ‘*Ca.* B. cookevillensis’, which could potentially allow multiple bacteria to pass through a gap at once [1].

Another possible scenario for entry of intact BCVs into the nucleus is that the vacuole has entered into the perinuclear space via the endoplasmic reticulum, which is continuous with the outer nuclear membrane, and either invaginates the inner membrane or enters through breaks in the inner membrane into the nucleus. Although indentation of the lamin-lined inner membrane occurs, as in Fig. 7a-b, invagination would result in the nuclear lamina surrounding the BCV and becoming continuous with the lamin-stained inner membrane (as in Additional file 4). Indirect immunofluorescent staining of infected HeLa cells with antibody to nuclear pore protein Nup62 provided evidence that the BCV is not between the nuclear membranes, nor is it tightly surrounded by Nup62-labeled nuclear envelope (Additional file 1: Figure S3). A Z-stack of confocal images and three-dimensional rotation depict the ‘*Ca.* B. cookevillensis’ vacuoles completely surrounded by the nuclear pore-stained nuclear envelope (Additional file 6).


**Additional file 6:** Confocal Z-stack and 3D rotation video (mp4 file) of HeLa cell nuclei stained with monoclonal antibody to nuclear pore protein Nup62 and containing DAPI-stained bacteria. (MP4 17560 kb)


### Entry of ‘*Ca.* Berkiella cookevillensis’ bacteria-containing vacuoles into the nucleus excludes cytoplasmic components

**Fig. 8:**
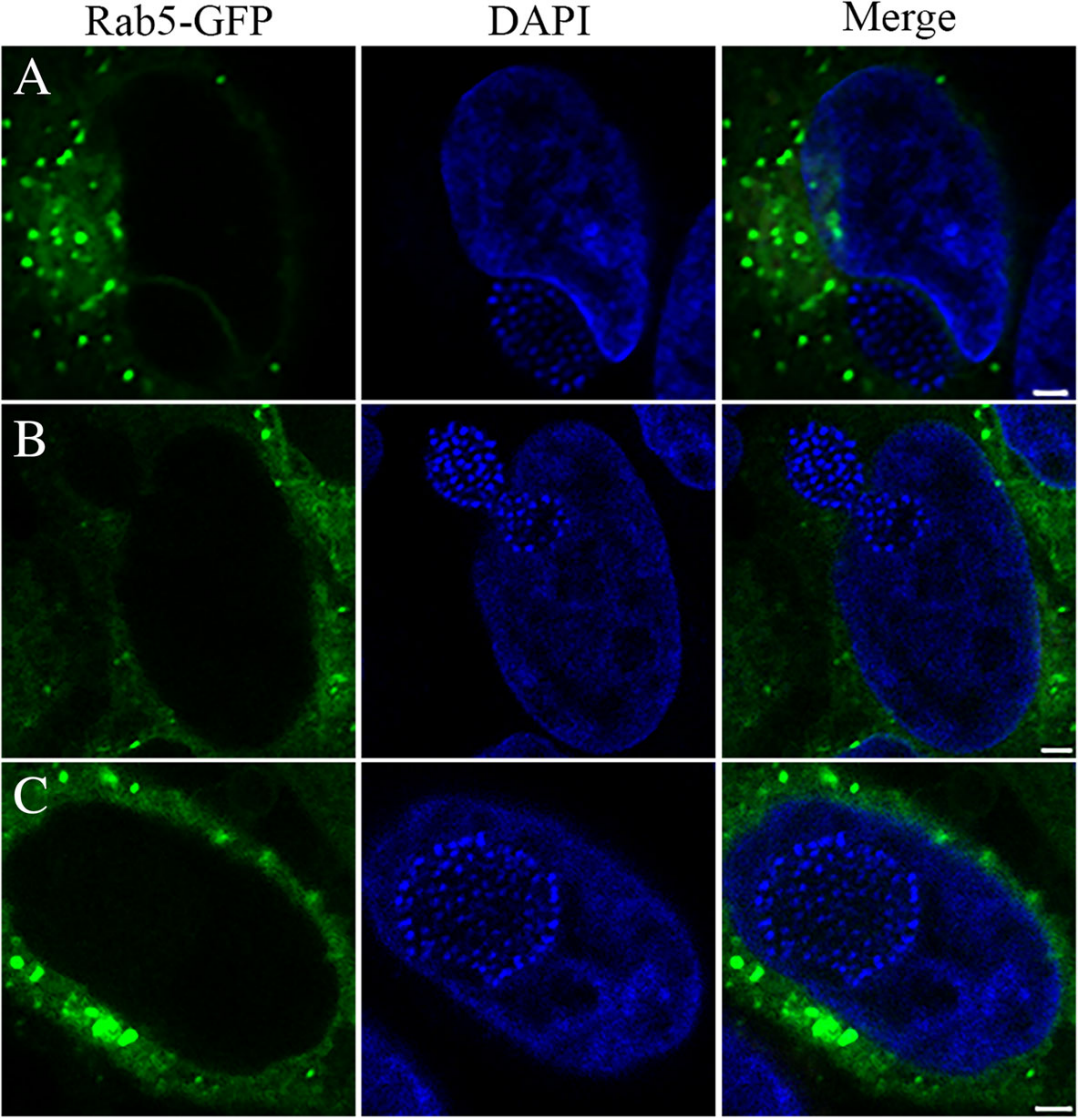
Exclusion of cytoplasmic markers during ‘*Ca.* Berkiella cookevillensis’ nuclear invasion of HeLa cells. HeLa cells were transduced to express Rab5a-GFP (green) prior to CC99 infection. In these images Rab5a-GFP not associated with early endosomes was used as a cytoplasm marker. Cells were infected at an MOI of 200 and incubated for 24 h. HeLa cell and bacterial DNA were stained with DAPI (blue). Image series demonstrates **a** CC99-containing vacuole in close proximity to the host nucleus, **b** invading the host nucleus, and **c** inside the host nucleus. Throughout the nuclear invasion process, the cytoplasmic marker Rab5a-GFP was excluded from the nucleus, nor was there any DAPI-stained chromatin in the cytoplasm. Bars, 2 μm

During characterization of the ‘*Ca.* B. cookevillensis’ vacuole, we determined whether Rab5a-GFP, an early endosome marker, was associated with the BCV. While we did not observe Rab5a-GFP associate with the BCV, the diffuse Rab5a-GFP throughout the cytoplasm provided an excellent cytoplasmic stain. Additionally, Rab5a-GFP does not passively diffuse through the nuclear pore complex, which often occurs with low molecular weight proteins like individual GFP molecules [20]. Using the diffuse Rab5a-GFP as a cytoplasmic marker, we observed that cytoplasm was present between the nuclear envelope and BCV when the vacuole was pressing against the nuclear envelope (Fig. 8a). However, when the BCV was traversing the nuclear envelope, no diffuse Rab5a-GFP was identified between the bacteria-rich vacuole and the nucleus (Fig. 8b). Furthermore, in images of the ‘Ca. B. cookevillensis’ BCV either crossing the nuclear envelope or within the nucleus, no Rab5a-GFP was detected in the nuclear compartment (Fig. 8b-c). The DAPI-stained nucleic acids also remained in the nucleus and were not observed in the cytoplasm during invasion by the BCV. The nuclear exclusion of Rab5a-GFP during the duration of the ‘Ca. B. cookevillensis’ BCV nuclear invasion suggests that there is a tight association of the bacteria-rich vacuole with the nuclear membranes. Furthermore, it indicates that the integrity of the nuclear envelope is maintained throughout the invasion process, suggesting a unique mechanism of entry not previously described for other intranuclear bacteria by which the bacteria pass through the nuclear envelope while enclosed in a membrane-bound vacuole.

## Discussion

We have previously described the discovery and identity of the novel bacteria, ‘*Ca.* Berkiella cookevillensis’ and ‘*Ca*. Berkiellea aquae’, as intranuclear bacteria in the amoebal host *A. polyphaga* [1, 21]. Here we report ‘*Ca.* B. cookevillensis’ as a bacterium associated with the nuclei of mammalian cell lines, including human cells. The bacterium appears to enter nuclei of both phagocytic and nonphagocytic cell lines enclosed in a vacuole. In all host cell lines that we treated, which includes amoeba, as well as phagocytic and nonphagocytic human and murine cell lines, ‘Ca. B. cookevillensis’ replicates and appears to fill the nucleus, eventually lysing the host cell. Infection of the amoebal host typically resulted in 100% infection and co-localization with nuclei within 48 h, resulting in host cell lysis, whereas in human cell lines, nuclear association was not observed to be above 65%. In the human cell lines, we also observed increases in numbers of bacteria within cytoplasmic vacuoles, signifying that association with the nucleus was not necessary for bacterial replication in these cells. The intracellular association of bacteria and amoebae is thought to have led to the evolution of intracellular pathogens of humans and other vertebrates [22], and it is an interesting possibility that the differences between infectivity for amoebae and human cells could reflect an early stage of evolution for ‘Ca. B. cookevillensis’ as a human pathogen. ‘Ca. B. cookevillensis’ were commonly surrounded by a lucent space indicative of an extracellular matrix and were within an evident vacuole. However, occasionally bacteria that appeared to be intranuclear were not contained within a vacuole, and it is as yet unclear whether a membrane surrounds bacteria early in the infection process.

Recent descriptions for nuclear invasion mechanisms include the escape from a phagosome by *Holospora* spp. and *Nucleicultrix amoebiphila* followed by either the use of a specialized actin-binding invasion tip or entry during nuclear envelope disintegration during mitosis [3]. Another mechanism described for nuclear symbionts of *Euglena* is the fusion of the phagosome with the outer nuclear membrane in which the bacteria are released into the perinuclear space and subsequently invaginate the inner nuclear membrane to enter the nucleus [16]. ‘*Ca.* B. cookevillensis’ appears to employ a mechanism that differs from those previously described. Although it is as yet unclear whether ‘Ca. B. cookevillensis’ escapes its initial phagosome and re-enters another membrane compartment within the cytoplasm, we typically observe bacteria enclosed within a membrane in TEMs and distinctly clustered bacteria in immunostaining. Immunostaining and confocal microscopy suggest that the bacteria traverse the nuclear envelope as an intact vacuole, providing evidence that ‘Ca. B. cookevillensis’ is able to enter the nucleus within a membrane vacuole. Furthermore, lamin staining indicates that a lamin-lined membrane has not formed around the bacteria as a result of a fusion-reforming event. Additionally, vacuoles appear to be pinched and elongated as they cross into the nucleus through breaks in the nuclear lamina (Figs. 7c and 8b). These breaks do not result in the entry of other cytoplasmic contents into the nucleus nor the entry of detectable chromatin into the cytoplasm as has been reported during Parvovirus entry into the nucleus [23].

Although the TEM images depict ‘*Ca.* B. cookevillensis’ within a membrane in the nucleoplasm, the two-dimensionality of the images cannot exclude the possibility that the image represents a cross section of a cell in which the BCV is invaginated into the nucleus. Many of the confocal images also support an invagination or ruffling of the host nuclear envelope around the BCV. ‘Ca. B. cookevillensis’ possesses the genes for a type IV secretion system, and although type III secretion systems are most often associated with plasma membrane ruffling, the type IV secretion system of *B. pseudomallei* has been implicated in ruffling of the nuclear envelope [10, 24].

Three scenarios might result from the ruffling and invagination of the nuclear envelope. In one scenario, both membranes are invaginated such that the BCV is surrounded by the nuclear envelope. In the second scenario, the BCV enters the perinuclear space through the endoplasmic reticulum and subsequently invaginates only the inner nuclear membrane. Association of ‘Ca*.* B. cookevillensis’ with endoplasmic reticulum is currently being studied in our laboratory. ‘Ca. B. cookevillensis’ is related to *L. pneumophila*, which subverts endoplasmic reticulum membrane for its vacuole [25]. Other bacteria, including *Brucella* and *Chlamydia* either fuse or interact with the endoplasmic reticulum membrane [25, 26]. If the BCV was localized to the perinuclear space, then the BCV should be closely surrounded by the nuclear membranes and continuous with the nuclear envelope. While some examples of lamin protein immunostaining show the lamina around the BCV, other examples depict BCVs without any evident lamin staining (Figs. 6 and 7b, Additional file 5), seemingly indicating the lack of inner nuclear membrane surrounding the bacteria. Even if a very deep invagination were to obscure the continuity of the invaginated membrane with the nuclear envelope, the lack of lamin staining suggests the ‘Ca. B. cookevillensis’ vacuole is not surrounded by the inner nuclear membrane. Similarly, immunostaining of nuclear pore proteins does not suggest that nuclear envelope tightly surrounds the BCV (Additional file 6). TEM images indicate that a single membrane encloses the bacteria with chromatin evident between the BCV and the inner nuclear membrane (Figs. 1b, 2a and 4b). However, there is often a close association of the BCV membrane with the nuclear membranes, indicating a potential interaction between the BCV and nuclear envelope (Fig. 4b). A close proximity of the BCV to the nuclear envelope is also visible in the majority of confocal images and videos. This association may be the result of either a fusion or interaction of those membranes or a consequence of the limited intranuclear space for the replicating bacteria.


**Additional file 5:** Confocal Z-stack video (mp4 file) of Fig. 7a with bacteria in HeLa cells stained with DAPI and anti-lamin A/C antibody, followed by the Z-stack with lamin immunostaining only. (MP4 14061 kb)


The third scenario would be for the BCV to cross both nuclear membranes and reside within a BCV surrounded by the chromatin. The lack of both lamin and nuclear pore staining around the BCV suggests this as a possibility, although it should be noted that none of the three scenarios excludes the occurrence of the other two. Breaks in the lamina of the inner nuclear membrane are evident in Figs. 6 and 7 (and Additional files 4 and 5). Similar disruption of the nuclear envelope is implicated by nuclear pore staining (Additional file 1: Figure S3). Nuclear envelope breakdown occurs during mitotic division. The association of the ‘*Ca.* B. cookevillensis’-rich vacuole with nucleic acids following nuclear envelope disassembly during mitosis and subsequent reformation of the nuclear envelope upon completion of mitosis is an appealing mechanism for entry into the nucleus. However, the ability of these vacuoles to enter the nucleus in the terminally-differentiated U937 and THP-1 cells does not support the need for mitosis for intranuclear infection [27, 28]. Many intranuclear viruses may also rely on the mitotic nuclear envelope breakdown for entry; however, other viruses are known to cross the nuclear envelope by mechanisms that do not rely on mitosis, such as entry through nuclear pore complexes [29]. The 39 nm diameter size limit for translocation through the nuclear pore complex excludes these channels as a probable entry route for the ‘*Ca.* B. cookevillensis’ vacuole [19]; however, some viruses cross the nuclear envelope by inducing non-mitotic disruptions of the nuclear membranes [29]. Parvovirues are small RNA viruses that enter the nucleus and although their entry is not well understood, recent evidence suggests that binding of viral capsids to nuclear pore complex proteins induces changes in calcium efflux that subsequently activate protein kinase C (PKC). PKC activation then activates cyclin-dependent kinase-2 (CDK-2) which is further activated by caspase-3. These activation steps for PKC and CDK-2 are necessary for nuclear envelope disintegration during mitosis [23]. Analysis of the ‘Ca. B. cookevillensis’ genome [2] implicates several putative proteins with eukaryotic-like domains for PKC and CDK phosphorylation, as well as domains involved in caspase-3 activation and recruitment. As mentioned above, Parvovirus-induced breaks can result in leaking of components between compartments. The possibility that ‘Ca. B. cookevillensis’ entry into the nucleus, like viruses budding from the host cells, might subvert host ESCRT-III proteins to rapidly reseal the nuclear envelope after entry through bacterial-induced gaps also needs further exploration [30, 31].

The *dot/icm* genes of *Legionella* spp. and *C. burnetii* encode the components for a type IVB secretion system. ‘*Ca.* B. cookevillensis’ has 26 *dot/icm* genes with no homologous genes yet identified for *icmT, icmN/dotK, icmM/dotJ, or icmX*, although there are 7 hypothetical proteins in close proximity to the *dot/icm* gene cluster [2, 32–36]. Proteins encoded by *dot/icm* genes form a transport system through which effector molecules are translocated. These effectors are used to recruit host cell molecules to both form and maintain the membrane compartments for replication of *Legionella* spp. and *Coxiella burnetii*. Identification of ‘Ca. B. cookevillensis’ effector proteins will provide further insight into the bacterium’s cytoplasmic and nuclear trafficking.

Similar to ‘*Ca.* B. cookevillensis’, host cell trafficking to the nucleus for ‘*Ca.* Endonucleobacter bathymodioli’, an intranuclear bacterium of bathymodiolin mussels, is not yet understood. ‘*Ca.* E. bathymodioli’ has been observed as entering the nucleus from the cytoplasm as a single, rod-shaped bacterium, which then enters a developmental cycle involving elongation into a long filament that subsequently divides into shorter filaments and then into shorter, rod-shaped cells that fill the nucleus [37]. We did not observe any such developmental changes in ‘*Ca.* B. cookevillensis’ in the vacuoles of their host cells. Zielinski et al. [37] also reported that as ‘Ca. E. bathymodioli’ fill the nucleus, loss of nuclear chromatin occurs suggesting the chromatin may serve as a nutrient source for the replicating bacteria. For ‘Ca. B. cookevillensis’, as the BCV expands, TEM images suggest that chromatin is diminished (Figs. 2b and 3b), but the intravacuolar location of the bacteria may preclude them from utilizing chromatin directly. However, as similarly described for both ‘Ca. E. bathymodioli’ and *N. amoebiphila* [3, 37], we observed that as ‘Ca. B. cookevillensis’ fills the host cell, the host membranes disintegrate followed by release of the bacteria. Contrary to descriptions for the other intranuclear bacteria, before bacterial release from the cell, ‘Ca. B. cookevillensis’ begins to exhibit circular motility similar to *L. pneumophila* in cytoplasmic vacuoles [38], which increases over the next few hours before lysis, after which the bacteria remain motile for several hours following release. This motility is more pronounced in amoebae than in mammalian host cells. Whether this release from the host cell results from mechanical disruption of the membrane or effectors from the bacteria is as yet unknown.

Nucleomodulins of bacteria have been described as a strategy for bacteria, particularly intracellular bacteria, to subvert the host processes by interfering with nuclear chromatin and proteins. Among these nucleomodulins are proteins with ankyrin repeats, a eukaryotic motif involved in protein-protein interactions. These protein motifs have been found in intracellular bacterial pathogens including *Rickettsia* spp. as well as *C. burnetii* and *Legionella* spp. [39, 40]. In the intracellular bacterium *Ehrlichia chaffieensis*, the anykyrin-repeat protein p200 binds to promoter regions in host cell DNA suggesting it may affect host cell transcription [41]. The ‘*Ca.* B. cookevillensis’ genome possesses several sequences encoding putative proteins with ankyrin-repeats, which could also be explored as potential effectors in the nucleus. Both *C. burnetii* and *L. pneumophila* encode effectors that translocate to the nucleus. These effectors modulate histone and chromatin structure as well as affect transcription of genes involved in host defense [34]. If a bacterium requires effector protein interaction with host nucleic acids, residing in the nucleus may provide the bacterium with greater accessibility to host chromatin.

As noted by Schulz and Horn in their review of intranuclear bacteria [3], genomic comparisons, as well as transcriptomic and proteomic approaches will aid in understanding nuclear invasion mechanisms, although the obligate nature of this bacterium has thus far limited genetic studies. In all cell types treated with ‘*Ca.* B. cookevillensis,’ BCVs co-localize with the nucleus by 24 h p.i., often dotting the nucleus with multiple BCVs. We are currently characterizing the membranes that ‘*Ca.* B. cookevillensis’ acquires as it traffics to the nucleus, which will help to understand its nuclear association and entry, whether this bacterium enters host nuclei by deeply invaginating the nuclear envelope and surrounding itself with membranes or by enclosure in a membrane that remains intact as it traverses the nuclear envelope from the cytoplasm. Either association could be evolutionarily advantageous for a bacterium to evade degradation by a host cell. ‘Ca. B. cookevillensis’ thus may not only employ an as yet undescribed strategy to invade the host nucleus, but this bacterium is also without precedent in its ability to overtly use the nucleus as a growth niche, exhibiting high frequencies of infection and significant increases in numbers of bacteria within mammalian cell nuclei.

## Conclusion

The bacterium ‘*Ca.* B. cookevillensis’ associates with the nuclei of amoebal and mammalian host cells. Initially the bacteria appear within cytoplasmic vacuoles of their host cell, and numbers of the bacteria increase within the vacuoles. The bacteria-containing vacuoles associate with the host nucleus and invaginate the nuclear membranes, either forming a deep pocket or entering as an intact vacuole into the nucleoplasm. Replication of the bacteria in association with the nuclei results in host cell lysis. This novel bacterium provides new insights into alternative strategies for survival in a range of host cell types.

## Methods

### Growth of *‘Ca.* Berkiella cookevillensis’ in cell lines

Bacterium ‘*Ca.* B. cookevillensis’ (strain CC99) was cultivated in *Acanthamoeba polyphaga* (ATCC 30461; American Type Culture Collection, Manassas, VA, USA) as previously described [1]. The human monocytic cell lines U937 (CRL-1593.2, ATCC) and THP-1 (TIB-202, ATCC) were grown in monolayer or suspension at 37 °C and 5% CO_2_ in complete RPMI medium [RPMI 1640 (Sigma-Aldrich, St. Louis, MO USA) supplemented with complement-inactivated 10% fetal bovine serum (Atlanta Biologicals, Atlanta, GA USA)]. The human epithelial-like HeLa (CCL-2.2, ATCC) and murine macrophage RAW 264.7 (TIB-71, ATCC) cell lines were grown in monolayer at 37 °C and 5% CO_2_ either in complete RPMI medium or in a Dulbecco’s Modified Eagle Medium (MEM)/Ham’s F-12 50:50 mix with 10% complement-inactivated fetal bovine serum and 0.1% MEM vitamins (Corning, Corning, NY, USA). For infectivity studies, amoebae, human monocytic, and murine cell lines were plated in 6-well tissue culture plates at a concentration of 5 × 10^5^ cells per well. Amoebae and adherent mammalian cell lines were incubated overnight to allow adhesion to the bottoms of the wells. HeLa cells were plated at a density of 3.5 × 10^4^ cells/mL for 48 h prior to infection. U937 and THP-1 cells were differentiated into macrophage-like cells using 200 nM phorbol myristic acid (PMA) and incubated for 72 h prior to treatment with bacteria. For fluorescent in situ hybridization and immunostaining experiments, sterile glass coverslips were added to wells prior to addition of cells. For infections, bacteria from 4 to 5 day lysates of *A. polyphaga* co-cultures were enumerated by epifluorescent microscopy (Olympus BX-60, Norfolk, VA, USA) after passing lysates through 5 μm PVDF filters to remove amoebal cells (Millipore-Sigma, Billerica, MA, USA) and staining of bacteria in the filtrate with 4′-6 diamino-2 phenylindole (DAPI), followed by filtration onto Whatman 0.1 μm black nucleopore filters (GE Healthcare, Pittsburgh, PA) for counting. Bacteria were added to host cells at a multiplicity of infection (MOI) of 1 for amoebae and 10–200 for mammalian cells. The MOI chosen was dependent upon whether the mammalian cell was phagocytic and the time at which the infected cells were to be visualized by microscopy. Filtered lysates of uninfected amoebae were used for control treatments. At 1 h post infection (p.i.), the medium in each well was removed, and each well was washed three times with fresh medium to remove extracellular bacteria. Cells were treated with 75 μg/mL of gentamicin for 1 h as previously described [42] followed by washing with medium as above. This was designated as the *t* = 0 time point.

For harvesting cells for quantitative real-time PCR and transmission electron microscopy, media from triplicate bacteria-treated and control wells for each cell type were gently aspirated into individual sterile tubes. *A. polyphaga* were dislodged by gentle tapping. Adherent mammalian cells were dislodged by trypsinization and gentle cell scraping. For qPCR, cells from each well were added to the tube containing the respective medium from that well. The cells in each tube were lysed by passage through a 27-gauge needle. A 1-mL aliquot of each suspension was centrifuged at 8000 *xg* for 10 min and the resulting pellet frozen at − 80 °C for qPCR analysis. Cells seeded on coverslips were fixed with 4% paraformaldehyde for 10 min for fluorescent in situ hybridization or immunostaining.

### Quantitative real-time PCR

DNA was extracted from frozen pellets of lysates harvested at selected intervals using the DNeasy Blood and Tissue kit (Qiagen, Valencia, CA, USA). Quantitative real-time PCR (qPCR) was performed using primers designed for the 16S rRNA gene sequence of ‘*Ca.* B. cookevillensis’ to ensure its specific detection [43] (Additional file 1: Table S1). Each 25 μL reaction contained 1X iQ SYBR Green supermix (BioRad, Hercules, CA, USA), 0.22–0.3 μM of each primer, and DNA extracted from lysates. qPCR was performed with the BioRad CFX96 real-time detection system using the following program: initial denaturation (95 °C for 3 min), followed by 40 cycles of denaturation (95 °C for 45 s), annealing (50 °C for 30 s), and extension (72 °C for 30 s), with a final extension (72 °C for 5 min). The specificity of the reactions was confirmed with melting curve analysis from 50 °C to 72 °C with detection every 0.5 °C. Bacterial concentration was estimated as genomic equivalents (GE) using specific primers for 16S rDNA, as genomic sequencing has identified a single copy of the 16S rRNA gene in *‘*Ca. B. cookevillensis’ [2]. Five- or ten-fold dilutions of a plasmid containing a single copy of the 16S rRNA gene [1] were used to generate a standard curve. For each qPCR plate, standards, samples, and negative controls were run in triplicate and results are representative of at least two independent experiments. Validation characteristics of the standard curves for the *‘*Ca. B. cookevillensis’ primers are shown in Additional file 1: Table S2.

### Fluorescence in situ hybridization (FISH)

FISH staining of cells on paraformaldehyde-fixed coverslips was performed using a 6-FAM-conjugated probe targeting the 16S rRNA of ‘*Ca.* B. cookevillensis’ and two helper probes upstream and downstream of the probe site to enhance probe binding [44] (Additional file 1: Table S3). FISH probes were designed by Ribocon (Bremen, DE). Candidate in situ hybridization probes were evaluated and selected using the ARB software package (http://www.arb-home.de) based on the SILVA SSU Ref NR 115 dataset of about 480 K nearly full length and non-redundant SSU rDNA sequences. For quality assurance, specificity of selected probes was confirmed using the complete SILVA Parc database [45]. The eubacterial antisense probe NON338FAM (probeBase accession pB-243) was used as a control for autofluorescence using the same conditions as for the ‘Ca. B. cookevillensis’ probe set described below [46]. All probes were synthesized by Eurofins MWG Operon (Huntsville, AL, USA). Prior to hybridization, cells on coverslips were dehydrated in an alcohol series (50, 80, and 100% ethanol for 3 min each at room temperature). Hybridization was conducted at 46 °C for 3 h with 3.6 ng/μL of the probe and each helper probe in hybridization buffer [900 mM NaCl, 20 mM Tris-HCl (pH 8.0), 10% formamide, 0.01% SDS]. Hybridization was followed by a brief wash [450 mM NaCl, 20 mM Tris-HCl (pH 8.0), 0.01% SDS] followed by a 25-min wash in the same buffer at 48 °C. Coverslips were rinsed with distilled water, air dried, and mounted onto slides using Vectashield with 1.5 μg/mL DAPI (Vector Laboratories, Burlingame, CA, USA). Cells were visualized by confocal microscopy. Probe specificity was determined by hybridizing the probes with *‘Candidatus* Berkiella aquae’ strain HT99, *Legionella pneumophila* AA100, and *E. coli* ATCC 25922*.*

### Confocal microscopy

Confocal microscopy was performed using a Zeiss AxioObserver microscope with LSM700 confocal module and Plan-Apochromat 63x/1.40 Oil DIC M27 objective. Pinholes were set so that section thickness was the same for all channels and ≤ 1 Airy unit, which was commonly 41 μm. For imaging, the excitation wavelength for DAPI was 405 nm; for TRITC, 555 nm, and for 6-FAM, Alexa Fluor 488, emGFP, and FITC fluorophores, 488 nm. The Smart Setup function of the ZEN 2012 imaging software (Black Edition, Carl Zeiss Microscopy, Thornwood, NY, USA) was used to assign the optimal filter and beam splitter settings for each laser. Furthermore, immunofluorescent images were obtained at a 512 X 512-pixel, 12-bit image format typically using a 12.6 μs pixel dwell time and line average 2. The FISH stain images were obtained at a 512 X 512-pixel, 8-bit image format with a 50.4 μs pixel dwell time. The laser power, gain, and single channel thresholds were set manually and retained for all samples in an experiment. Imaging and fluorescence intensity measurements were performed with ZEN 2012 software. Using ZEN 2012 software, Z-stacks were obtained by acquiring 30 images at a slice interval of 0.558 μm and 1.58 μs pixel dwell with the Z-stack function. The Ortho View feature of ZEN 2012 software was used to display the Z-stack of images in an orthogonal view.

### Transmission electron microscopy (TEM)

For TEM, cells exposed to bacteria in 6-well plates were harvested at selected time points, fixed in 2.5% glutaraldehyde in 0.1 M phosphate buffer, pH 7.4, for 2 h, followed by post-fixation with 2% (*w*/*v*) aqueous osmonium tetroxide (EMS, Hatfield, PA, USA) for 2 h at room temperature. Fixed specimens were dehydrated in an ascending ethanol series from 30 to 100% with final dehydration in propylene oxide (EMS). Samples were embedded in Epon-Araldite (EMS) and 25 nm sections were stained with 5% uranyl acetate and lead citrate (Ted Pella, Inc., Redding, CA, USA) [47]. Microscopy was performed with a Hitachi H-7650-II (Schaumburg, IL, USA) transmission electron microscope.

### Cell staining and enumeration

Following infection, control and infected cells were enumerated with a hemacytometer. Cells were visualized by brightfield microscopy on an Olympus BX-60. At *t* = 0, 24, 48, and 72 h, co-cultures were also stained with the LIVE/DEAD BacLight viability stain (Life Technologies, Grand Island, NY, USA) according to the manufacturer’s recommendations and observed under 1000X magnification using either a Leica TCS SL confocal laser scanning microscope (Buffalo Grove, IL, USA) or Nikon D-Eclipse C1 confocal microscope (Melville, NY, USA) equipped with differential interference contrast (DIC) optics and argon and helium-neon lasers.

### Immunofluorescent staining

Paraformaldehyde-fixed cells on coverslips were washed three times with Dulbecco’s phosphate-buffered saline (DPBS, Sigma-Aldrich), then cells were permeabilized, blocked, and autofluorescence quenched by incubation for 1 h at room temperature with 0.1% Triton X-100 or 0.5% Tween 20 (*v*/v), 5% normal goat serum (v/v), and 0.3 M glycine in DPBS. Custom anti-serum against ‘*Ca.* B. cookevillensis’ was generated by immunizing rabbits with ‘Ca. B. cookevillensis’ (Cocalico Biologicals, Stevens, PA, USA) isolated from *A. polyphaga* on Renografin gradients as previously described [1]. The custom anti-serum was used at a 1:100 dilution in 0.05% Tween 20 and 5% goat serum in DPBS. Cells were incubated with anti-serum at 4 °C overnight. The negative control was incubated with antibody buffer only. Samples were washed four times with 0.05% Tween-20 (v/v) for 5 min each. The secondary antibody staining used either goat anti-rabbit IgG, F (ab’)_2_-TRITC (Santa Cruz Biotechnology, Dallas, TX, USA) or goat anti-rabbit IgG (H + L)-Alexa Fluor 647 (Invitrogen, Carlsbad, CA, USA). Samples were washed four times with 0.05% Tween-20 (v/v) in DPBS for 5 min each, and coverslips were mounted using Vectashield with 1.5 μg/mL DAPI and observed by confocal microscopy. For staining of the nuclear lamin, mouse monoclonal antibody (IgG1) to recombinant human lamin A/C [MANLAC1 (4A7); Developmental Studies Hybridoma Bank (DSHB), University of Iowa, USA] was used at a 4 μg/mL concentration in 0.05% Tween 20 and 5% goat serum in DPBS. MANLAC1 (4A7) was deposited to DSHB by Morris, G.E. as DSHB Hybridoma Product MANLAC1 (4A7). Nuclear pore staining was performed with monoclonal antibody to the nucleoporin Nup62 (IgG1, Clone 414, EMD Millipore, Burlington, MA, USA) with the same concentration and conditions as those for the lamin A/C. Following treatment and fixation, cells on coverslips were incubated with the supernatant at 4 °C overnight, followed by secondary goat anti-mouse IgG, F (ab’)_2_-FITC (Santa Cruz Biotechnology) using conditions described above.

### Rab5a-GFP transduction of HeLa cells

Expression of Rab5a-GFP was performed by transducing HeLa cells with CellLight™ Early Endosomes-GFP, BacMam 2.0 (Thermo Fisher Scientific, Waltham, MA, USA) prior to bacterial infection. Briefly, HeLa cells were plated onto glass coverslips at a density of 7.5 × 10^4^ cells/mL. After 24 h, the BacMam 2.0 reagent was added to the HeLa cells at a concentration of 30 particles per cell, and incubated for an additional 24 h, after which they were infected with bacteria as previously described.

### Data analysis

A two-tailed, unpaired Student’s *t* test (Analysis ToolPak in Microsoft Excel 2016) was used to compare means between control and treatment groups. Differences were considered statistically significant at *p* ≤ 0.05. All reported values are means and standard error (SE) of triplicate samples from at least two independent experiments.

## Additional files

Additional file 1: A pdf file with tables of PCR and FISH primers and graphs of bacterial growth in *A. polyphaga* and human cell lines. (PDF 432 kb)

## Abbreviations

BCV: Bacteria-containing vacuole
CC99: Strain of *‘*Ca. B. cookevillensis
CDK-2: Cyclin-dependent kinase-2
DAPI: 4′,6-Diamidino-2-Phenylindole, Dihydrochloride
*dot/icm*: defective in organelle trafficking/intracellular multiplication
FAM: 6-carboxyfluorescein
FBS: Fetal bovine serum
FISH: Fluorescent in situ hybridization
FITC: Fluorescein isothiocyanate
GFP: Green fluorescent protein
IgG: Immunoglobulin G
MEM: Minimal essential medium
MOI: Multiplicity of infection
p.i.: post-infection
PKC: Protein kinase C
PMA: Phorbol 12-myristate 13-acetate
PVDF: Polyvinylidene fluoride
qPCR: Quantitative polymerase chain reaction
Rab5a: Ras-related protein 5a
SSU: Small subunit
TEM: Transmission electron microscopy
