# Supplementary material for: Infection and nuclear interaction in mammalian cells by ‘Candidatus Berkiella cookevillensis’, a novel bacterium isolated from amoebae
Source: BMC Microbiol. 2019 May 9;19:91. doi: 10.1186/s12866-019-1457-z (PMC6507137; doi:10.1186/s12866-019-1457-z)
Supplement: Supplementary file 6 — A pdf file with tables of PCR and FISH primers and graphs of bacterial growth in A. polyphaga and human cell lines. (PDF 432 kb) [file 12866_2019_1457_MOESM1_ESM.pdf]

Additional File 1: Supplemental Tables and Figures

Additional File 1: Table 1. Primers used for real-time quantitative PCR

Primer*	Target Bacterium	Sequence (5' to 3')	Bases	Tm (ºC)
**CC443F**	CC99	GTCTTGATGTTAATACC	17	44.8
**CC596R**	CC99	TCACATCTGACTTATCC	17	48.4

* Primers were designed for this study using PrimerQuest software from Integrated DNA Technologies and synthesized by Integrated DNA Technologies (Coralville, IA, USA). Primers were checked for specificity (most recent search July 2017) using the NCBI Primer-BLAST tool. The '*Ca.* Berkiella cookevillensis' (CC99) primer pair also targets 16S rRNA gene sequences reported for two uncultured bacterium clones (Accession numbers EU369126 and EU50792) which have 100% identity to the CC99 sequence and most likely represent the same bacterium. Primer CC443F also targets two other uncultured bacterial clone sequences. All other sequences in the database would have at least one mismatch per primer. Primers were tested for specificity using '*Candidatus* Berkiella aquae' strain HT99, *L. pneumophila* AA100, and *E. coli* ATCC 25922.

Additional File 1: Table 2. qPCR efficiency, amplification, slope y-intercept, and regression coefficient (R^2) values of the standard curves for qPCR amplification of '*Ca.* Berkiella cookevillensis' (CC99) 16S rDNA gene

Primer System	Efficiency (%)	Amplification	Slope	y-intercept	R^2 value
CC99 (CC443F– CC596R)	100.82	2.01	-0.303	13.06	0.987

Additional File 1: Table 3. FISH probes and helper probes used in this study.

Target Bacterium	Probe Name	Probe Sequence (5' to 3')*	Label
**'Ca. Berkiella cookevillensis' (CC99)**	CC832FAM<br>CCH796<br>CCH814<br>CCH850<br>CCH874	[6-FAM]CGATACCAGATGGTCTAA<br>CTCATAGTTTACGGCGTG<br>GCCACCTAACATCTAGTT<br>AACTTATCGCGTTAGCTG<br>TGCGGCCGTACTCCCCAG	5'-[6-FAM]
**Nonbinding Control**	NON338FAM†	[6-FAM]ACTCCTACGGGAGGCAGC	5'-[6-FAM]

* FISH probes and helper probes for '*Ca*. B. cookevillensis' (CC99) were designed by Ribocon (Bremen, DE) using the ARB software package (www.arb-home.de) based on the SILVA SSU Ref NR 115 dataset. Specificity of selected probes was confirmed using the complete SILVA Parc database. Probe CC832FAM also targets two 16S rRNA gene sequences reported for uncultured bacterium clones (Accession numbers EU369126 and EU50792) which have 100% identity to the CC99 sequence and most likely represent the same bacterium. One mismatch occurs with another uncultured bacterial clone (Accession JX227393). All other reported sequences have at least two mismatches. Probe CC832FAM binds in regions of low *in situ* accessibility (class V), and alterations in formamide concentrations and temperature did not produce a strong fluorescent signal when CC832FAM was used as the only probe. For this reason helper probes were employed and optimal hybridization conditions for signal strength with the probes were determined in experiments combining variations in both formamide concentration and temperature.

† Probe NON338FAM was used to control for background autofluorescence. Hybridization conditions were the same as those used for the CC99 probe set.

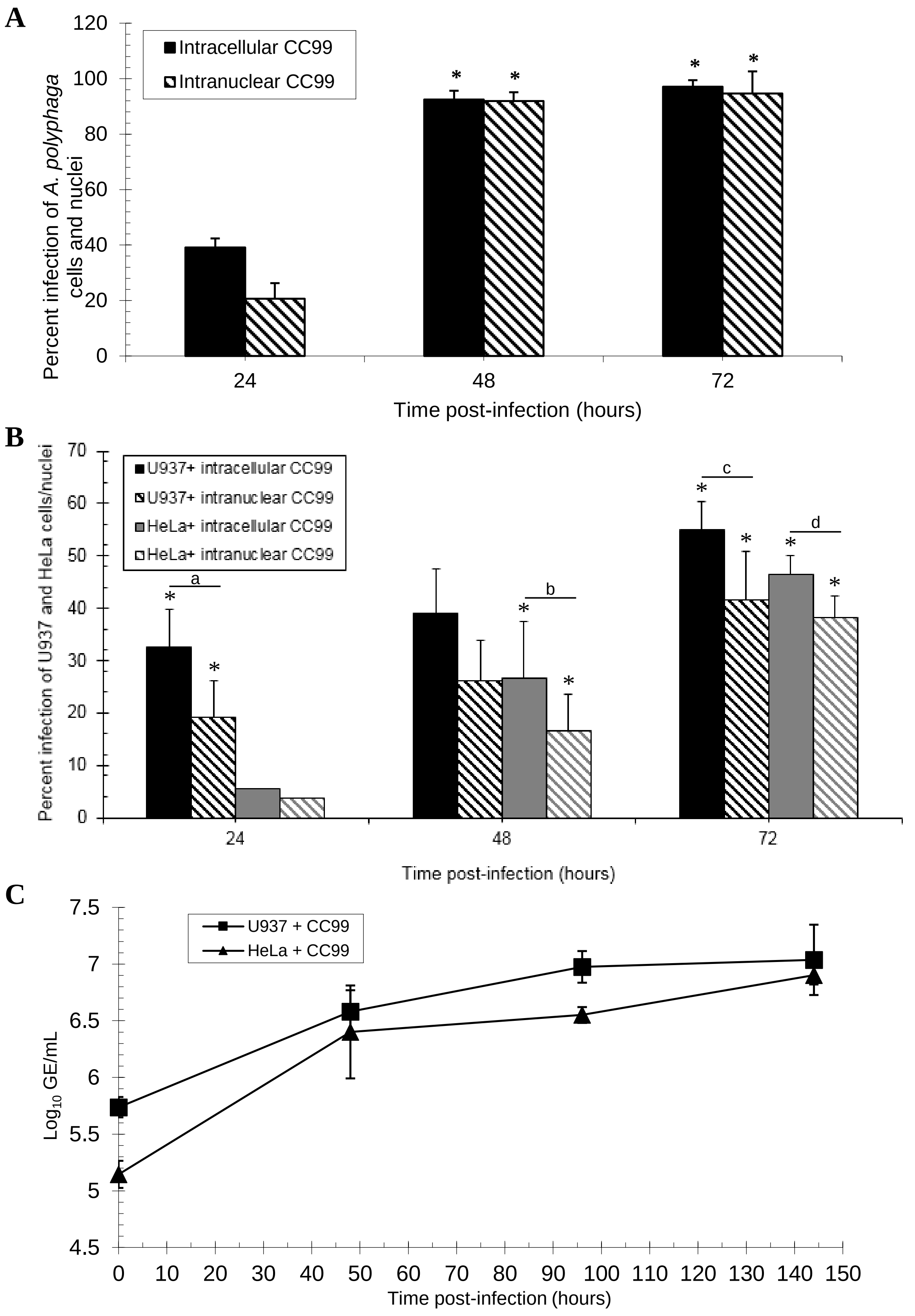
A
Intracellular CC99
Intranuclear CC99
Percent infection of A. polyphaga cells and nuclei
Time post-infection (hours)
B
U937+ intracellular CC99
U937+ intranuclear CC99
HeLa+ intracellular CC99
HeLa+ intranuclear CC99
Percent infection of U937 and HeLa cells/nuclei
Time post-infection (hours)
C
U937 + CC99
HeLa + CC99
Log10 GE/mL
Time post-infection (hours)

Additional File 1: Figure 1. '*Ca.* Berkiella cookevillensis' intracellular growth in eukaryotic cells.

(A) *Acanthamoeba polyphaga.* At selected time points post infection (p.i.), *A. polyphaga* cells infected with '*Ca.* B. cookevillensis' (CC99) at an MOI of 1 were stained for counting. Microscopic counts of at least 10 fields of cells from triplicate samples indicated that by 48 - 72 h p.i. over 95% of cells are infected with nuclear-associated bacteria, which is statistically, significantly greater (*) than at 24 h ($p \leq 0.05$). No significant increases in infection rates occur between 48 h and 72 h.

(B) Human PMA-differentiated U937 macrophage-like cells and HeLa epithelial cells.

At selected time points, differentiated U937 or HeLa cells infected with '*Ca.* B. cookevillensis' (CC99) at an MOI of 10 (U937) or 100 (HeLa) were stained for counting. Microscopic counts of at least 10 fields of cells from triplicate samples was performed. At 24 h p.i., percentages of infected cells and nuclear-associated bacteria for U937 cells are significantly greater than percentages for HeLa cells (a). Numbers of infected cells or nuclear-associated bacteria do not differ significantly, signifying the majority of host cells have bacteria co-localized with the nucleus. By 48 h, there are no significant increases in infection levels for U937 cells, but HeLa cell infections increased significantly from 24 h (b) with no significant differences between infected cells and bacteria co-localized to nuclei. At 72 h, percentages of infected U937 cells and nuclei increased from the 24 h time point (c), and percentages of infected HeLa cells and nuclear co-localization increased significantly from 48 h (d). Statistical significance was determined at $p \leq 0.05$ (*). After 72 h, cell lysis began to occur and no increases in infection were detectable. Infection levels did not rise above 65% of cells or nuclear-associated bacteria in any of the experimental trials.

(C) Growth of '*Ca.* B. cookevillensis' in U937 and HeLa cells as measured by qPCR. The bacterium increased in U937 cells by 1.25 $\log_{10}$ and in HeLa cells by 1.35 $\log_{10}$. Data represent means of at least two independent experiments performed in triplicate.

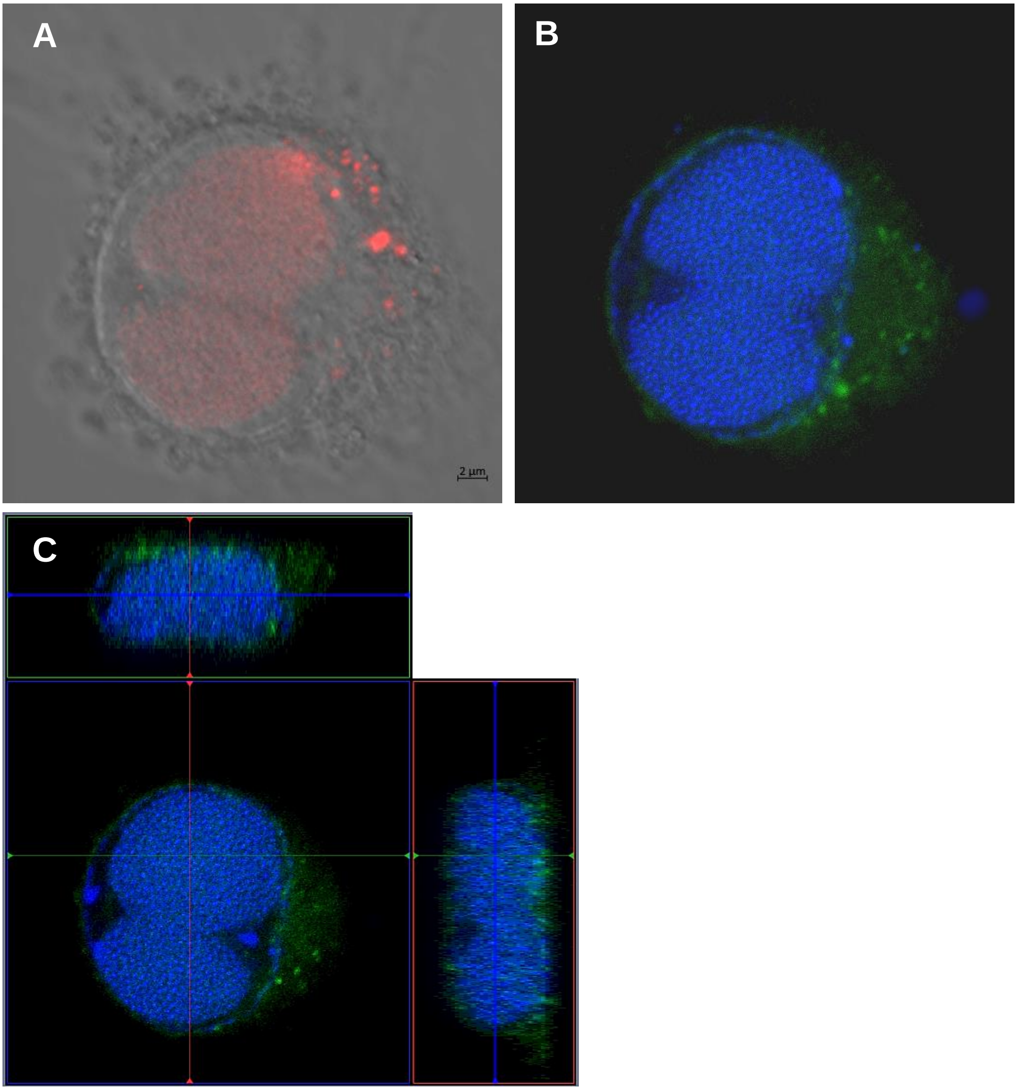

Additional File 1: Figure 2. '*Ca.* Berkiella cookevillensis' intracellular growth in murine RAW 264.7 macrophage-like cells.

(A) Murine RAW 264.7 macrophages with infected with '*Ca*. Berkiella cookevillensis' for 1 h at an MOI of 100, followed by gentamicin treatment for 1 h. Infection continued 36 h after which bacteria with cells and bacteria were stained with rabbit anti-serum to '*Ca*. B. cookevillensis' and an Alexa Fluor 647-conjugated secondary antibody (red). Confocal DIC image shows two bacteria-containing vacuoles within the nucleus.

(B) View of infected RAW 264.7 cell with DAPI-stained bacteria and chromatin (blue). Nuclear pore protein Nup62 was stained with monoclonal antibody and a FITC-conjugated secondary antibody. A nuclear pore-stained envelope (green) encloses both vacuoles.

(C) Orthogonal view centered on the upper bacterial vacuole shows that the nuclear pore staining (green) surrounds the DAPI-stained bacteria (blue).

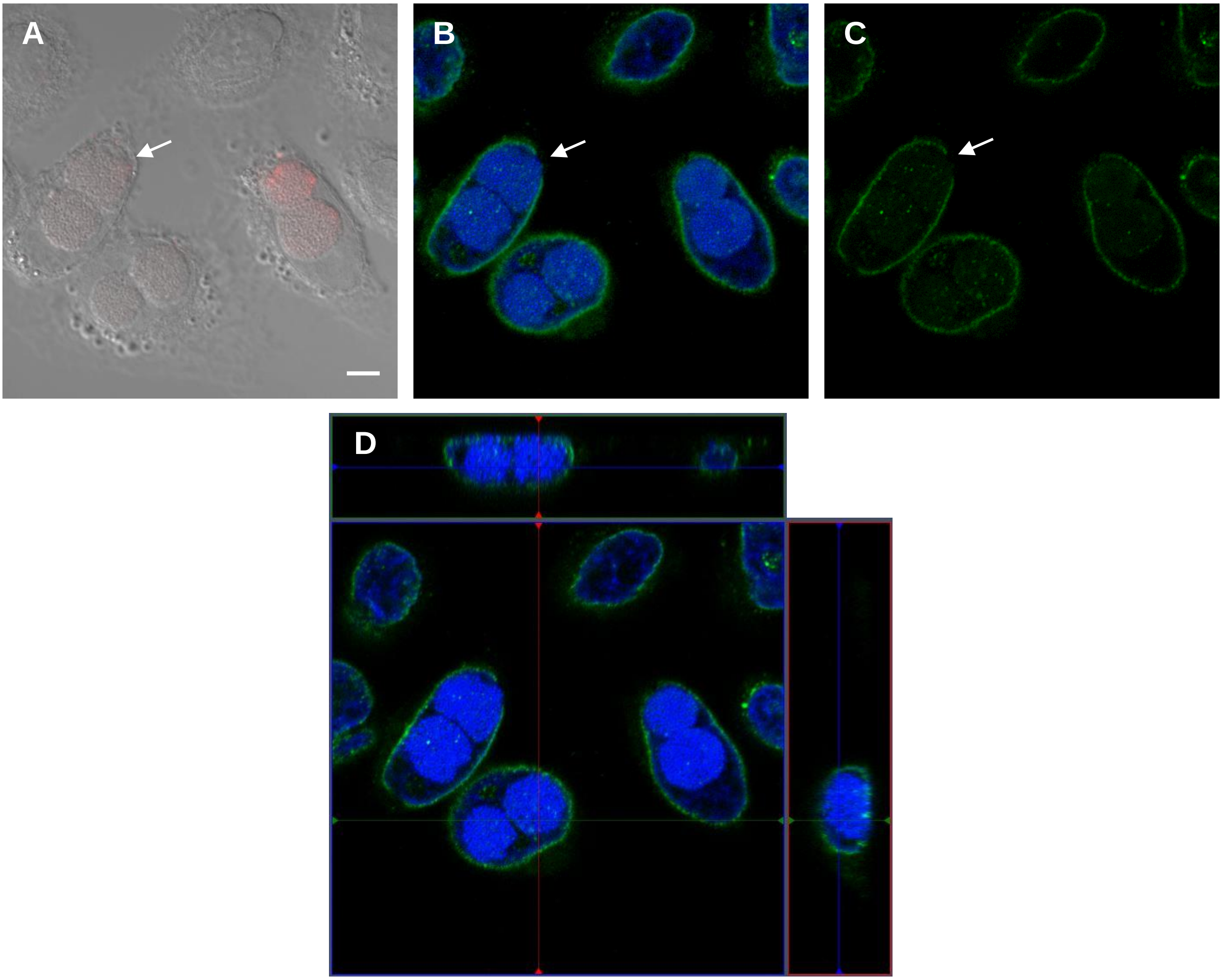

Additional File 1: Figure 3. Nuclear pore staining of HeLa cells infected with '*Ca.* Berkiella cookevillensis.'

(A) HeLa cells were infected with '*Ca*. Berkiella cookevillensis' for 1 h at an MOI of 200, followed by gentamicin treatment for 1 h. Infection continued for 36 h after which cells and bacteria were stained with rabbit anti-serum to '*Ca*. B. cookevillensis' and an Alexa Fluor 647-conjugated secondary antibody (red). Confocal DIC image shows 3 cells, each with two bacteria-containing vacuoles, within the nucleus. Arrow represents disruption of the nuclear envelope. Bar = 5 μm.

(B) '*Ca*. Berkiella cookevillensis'-infected HeLa cells stained with DAPI (blue) and monoclonal antibody for nuclear pore protein Nup62 and FITC-conjugated secondary antibody (green). Disruption of the nuclear pore staining is evident in the left and right nuclear envelopes (arrow).

(C) '*Ca*. Berkiella cookevillensis'-infected HeLa cells with staining for nuclear pore only highlighting the disruption of staining in the nuclear envelope (arrow).

(D) Orthogonal view centered on the two bacteria-containing vacuoles in the center cell. No disruption of the nuclear pore staining is visible in this view. See also Additional file 6.
